# Article series: from the first issue of Mem Inst Oswaldo Cruz (1909) to the present (2024)

**DOI:** 10.1590/0074-02760240250

**Published:** 2025-02-24

**Authors:** Adeilton Alves Brandão, Ana Carolina P Vicente, Ricardo Lourenço-de-Oliveira

**Affiliations:** 1 Fundação Oswaldo Cruz-Fiocruz, Instituto Oswaldo Cruz, Rio de Janeiro, RJ, Brasil

In 1906, during the XV International Congress of Medicine in Lisbon, Portugal, the “exotic pathology^1^ or colonial medicine” got a new designation: tropical medicine.^2^ The recommendation approved in that congress was indeed the formal recognition of the trend started with the creation of the first organisations dedicated to study the diseases of the tropics, *e. g.*, the Liverpool School of Tropical Medicine and the London School of Tropical Medicine and Hygiene (1899). It is also important to remind that all of the Tropical Medicine organisations created at the beginning of 20th had the explicit goal of supporting the European colonialism in Africa and Asia. It is in this historical context that Memórias do Instituto Oswaldo Cruz was officially established in 1907, and published the first issue in April 1909.

In the wake of the celebration events that mark the 115 years of the first issue of Memórias, we sought to view in retrospect what happened to the subject matters presented in eight articles published in the April 1909 issue and invited expert researchers in to present an update view on those “contributions to tropical medicine knowledge”.

The articles of the first issue described some aspects and technical challenges on tuberculosis, diphtheria, plague, malaria parasites, amoeba, and haematophagous insects such as anopheline vectors and horseflies.^3^ ^,^ ^4^ ^,^ ^5^ ^,^ ^6^ ^,^ ^7^ ^,^ ^8^ ^,^ ^9^ ^,^ ^10^ The discovery of the malaria causative agents (1880), serum therapy against diphtheria (1893) and plague (1895) and anopheline mosquitoes as the vectors of human malaria (1898) were still fresh and scientific production on these pulsating topics was growing in geometric proportions in international literature. Interestingly, these themes were among the most relevant public health problems and the most throbbing subjects in the so called tropical medicine in the first decade of 20th century in Brazil and worldwide. Not surprisingly, at the closing of the first quarter of 21st century tuberculosis, malaria, amoebiasis and vector control are still demanding responses from both researchers and public health officials in developing countries.

This article series about the first issue of Memórias is not only a “looking back” to 20th century challenges in tropical medicine but an opportunity to reflect on what lessons have we learnt and the mistakes we should not repeat 115 years later.

Despite being a century old journal, Memórias keeps going to incorporate innovations into its editorial practice. It is in the path to align itself with the open science and strives to make the researchers’ publishing journey a collaborative effort that is not only productive but also pleasant.
